# Viral miRNAs as Active Players and Participants in Tumorigenesis

**DOI:** 10.3390/cancers12020358

**Published:** 2020-02-04

**Authors:** Alessia Gallo, Vitale Miceli, Matteo Bulati, Gioacchin Iannolo, Flavia Contino, Pier Giulio Conaldi

**Affiliations:** 1 Department of Research, IRCCS ISMETT (Istituto Mediterraneo per i Trapianti e Terapie ad alta specializzazione), 90100 Palermo, Italy; vmiceli@ismett.edu (V.M.); mbulati@ismett.edu (M.B.); giannolo@ismett.edu (G.I.); fcontino@ismett.edu (F.C.); pgconaldi@ismett.edu (P.G.C.); 2 Scienze Mediche Chirurgiche E Sperimentali, Università degli Studi di Sassari, Piazza Universita, 07100 Sassari, Italy

**Keywords:** viral miRNAs, EBV, HHV-8, HPV, HCV, HBV, MCPyV

## Abstract

The theory that viruses play a role in human cancers is now supported by scientific evidence. In fact, around 12% of human cancers, a leading cause of morbidity and mortality in some regions, are attributed to viral infections. However, the molecular mechanism remains complex to decipher. In recent decades, the uncovering of cellular miRNAs, with their invaluable potential as diagnostic and prognostic biomarkers, has increased the number of studies being conducted regarding human cancer diagnosis. Viruses develop clever mechanisms to succeed in the maintenance of the viral life cycle, and some viruses, especially herpesviruses, encode for miRNA, v-miRNAs. Through this viral miRNA, the viruses are able to manipulate cellular and viral gene expression, driving carcinogenesis and escaping the host innate or adaptive immune system. In this review, we have discussed the main viral miRNAs and virally influenced cellular pathways, and their capability to drive carcinogenesis.

## 1. Introduction

According to the IARC (International Agency for Research on Cancer), 13% of new cancer cases worldwide in 2018 were the result of a chronic infection, most of which were caused by viruses [1]. The burden of viral infections in cancer, even if considered high, is still undervalued [2]. Viruses implement multiple strategies to pursue their final goals: viral survival, proliferation, and transmission. Moore and Chang masterfully emphasized that the event of cancer caused by viruses is a biological accident, since it does not increase transmissibility or enhance replication fitness [2]. Moreover, in the particular cases of immunosuppressed populations, cancers generated by tumor virus carcinogens have an increased incidence [3], suggesting the deep relationship between viruses and the immune system. Innate immune signaling shares many key effector proteins with tumor suppressor signaling, such as the p21 cyclin-dependent kinase inhibitor [4] and p53 [5]. This may imply the crucial role of tumor suppressor pathways in inadvertently placing the infected cell at risk for cancerous transformation [6,7]. One of the main roles in this process might be played by virally encoded miRNAs, ideal and non-immunogenic tools for viruses, able to modulate viral as well as host gene expression and lead to immune invisibility of infected cells [8]. V-miRNAs (v-miRNAs) seem to have a leading role in viral persistence and propagation, enacting different immune evasion strategies. V-miRNAs and host miRNAs can both regulate the expression of multiple host- and virus-derived transcripts [9]. An appealing theory suggests the use of v-miRNA orthologues of cellular miRNAs, with which they share a seed sequence and thus regulate the same targets. Still, among oncoviruses, only a few viral orthologues miRNAs have been discovered: kshv-miR-K12-11, which shows significant homology to cellular hsa-miR-155 [10]; kshv-mir-K12-10, which is a viral orthologue of hsa-mir-142-3p [11]; kshv-mir-K3, a homolog of hsa-mir-23 [12]; and ebv-miR-BART-5, which shows significant “seed” sequence homology to hsa-miR-18 [13].

**Figure 1:**
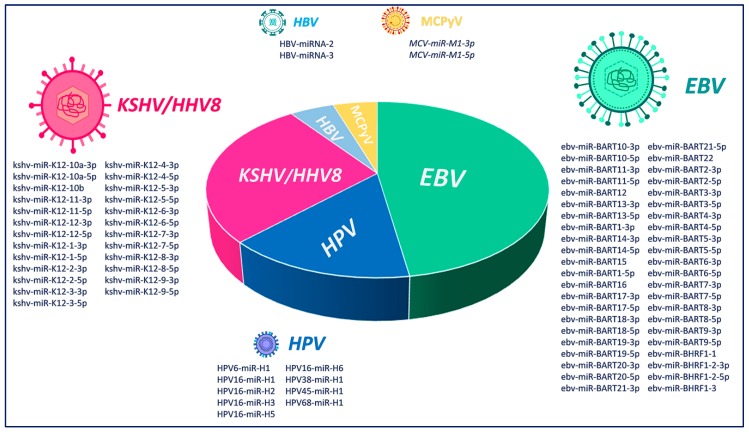
Graphical representation of the relative abundance of viral miRNA production by Epstein–Barr virus (EBV), Kaposi’s Sarcoma Herpesvirus/Human Herpesvirus-8 (KSHV/HHV8), Human Papillomavirus (HPV), Hepatitis C Virus (HCV), Hepatitis B Virus (HBV) and Merkel Cell Polyomavirus (MCPyV) viruses.

V-miRNAs have slowly evolved and adapted within their specific hosts. In fact, viral miRNA biogenesis involves only cellular factors, as no viral proteins have been described [9]. V-miRNAs are also exported via the exosomal route, rendering them able to enter into cells even at distant sites, thus allowing the virus to manipulate cellular and tissue immunity [14]. Considering that the survival ability of a virus depends on its capacity to escape host immunosurveillance [14], viruses encode multiple miRNAs that show immunomodulatory functions involved in the regulation of critical innate and adaptive immune mechanisms used by the host to defend himself [14]. Moreover, it has been described that v-miRNAs allow viruses to enter the latent phase of their life cycle and become undetected by the host’s immune system, with this being a further risk factor for cancer development [15]. Here we have examined the current knowledge of miRNAs encoded by six oncoviruses, Epstein–Barr virus (EBV), Kaposi’s Sarcoma Herpesvirus/Human Herpesvirus-8 (KSHV/HHV8), Human Papillomavirus (HPV), Hepatitis C Virus (HCV), Hepatitis B Virus (HBV) and Merkel Cell Polyomavirus (MCPyV) (Figure 1), and the virally influenced cellular pathways (Table 1) and their relationships with the immune system.

## 2. Epstein–Barr Virus (EBV)

The Epstein–Barr virus (EBV) is a ubiquitous lymphotropic gamma herpesvirus able to infect >95% of individuals during childhood and early adolescence. It usually causes an asymptomatic infection without significant illness, except some cases in which it may cause infectious mononucleosis [16]. After primary infection, EBV silently inhabits mainly the long-lived memory B cells of infected individuals. In immunocompromised patients, including organ transplant and AIDS patients, in southern Chinese patients, sub-Saharan African children, and other particularly susceptible groups, EBV is linked to a range of cancers and other disorders [16]. In the context of immunosuppression, post-transplant lymphoma, Hodgkin’s disease, African Burkitt’s lymphoma, and nasopharyngeal carcinoma are the malignancies most consistently and significantly associated with EBV [17]. The study of EBV and its potent growth-transforming action on infected lymphocytes associated with tumors points to specific interactions between environmental, genetic, and viral factors [18]. The EBV life cycle consists of a latent and lytic phase [19]. During the lytic cycle, EBV expresses its full set of viral genes [20]. In the lifelong latent phase, EBV infection establishes different transcription programs expressing a limited quantity of viral genes [21]. The pattern of EBV gene latency expression is essential for its genome maintenance and could be a key component of the puzzle of EBV-induced cancers [16,22]. V-miRNAs were detected for the first time in EBV [23] and, to date, a total of 44 mature miRNAs from 25 miRNA precursors have been encoded [24]. Recent evidence strongly suggests a role of EBV-encoded miRNAs in driving the initiation and progression of EBV-associated malignancies [8]. EBV encodes 44 miRNAs transcribed from two regions: the BamHI-A region rightward transcript (BART), with 22 miRNA precursors (ebv-mir-BART1-22) producing 40 mature miRNAs; and the BamHI fragment H rightward open reading frame 1 (BHRF1) clusters [25], with three miRNA precursors (ebv-mir-BHRF1-1, -2, and -3) producing four mature miRNAs [24]. As for the EBV genes, v-miRNA expression is infection-stage-dependent. BART miRNAs are transcribed in all stages of latency, although more associated with latency types I and II [26]. In contrast, BHRF1 miRNAs are amply expressed in type III latency, but nearly undetectable in latency types I and II [27]. All EBV-infection-associated human tumors display latency programs and related v-miRNA expression, spanning from latency I in BL, NK/T-cell lymphoma, and EBV aGC, to latency II in Hodgkin’s disease and nasopharyngeal carcinoma (NPC), and latency III in EBV-associated B lymphoma and Post-transplant lymphoproliferative disorder (PTLD) [28]. Several studies have pointed out that EBV miRNA clusters take part in tumor progression by targeting tumor-suppressing genes and repressing anti-proliferation genes. For example, ebv-mir-BART1 in NPC samples, is able to significantly up- and downregulate a number of genes fundamental for cell metabolism, including PAST1, PHGDH, DHRS3, ASS1, IDH2, PISD, UGT8, and LDHB [29]. Ebv-mir-BART5-5p plays an anti-apoptotic role by directly acting on the pro-apoptotic gene PUMA [30] and suppressing the p53 protein in various stomach cancer and NPC cell lines [31]. Other evidence has shown that ebv-mir-BART5-5p, together with ebv-mir-BART1-3p and ebv-mir-BART7-3p, promotes NPC cell metastasis by targeting the cellular tumor suppressor PTEN [32,33]. In BL, the regulation of PTEN by ebv-mir-BART6-3p promotes cell proliferation and inhibits cell death [34]. In addition, in NPC cells, ebv-mir-BART22 promotes metastasis by targeting N-myc downstream regulated gene 1 (NDRG1), known to be a tumor suppressor [35]. In gastric cancer cell lines, ebv-mir-BART4-5p reduces Bid expression, leading to reduced apoptosis [36]. In nasopharyngeal carcinoma cells, ebv-mir-BART22 has been shown to have proliferative and invasive abilities through the regulation of MAP3K5 [37]. Ebv-mir-BART20-5p has been found to shorten apoptosis, strengthen cell growth, and contribute to carcinogenesis of EBVaGC by directly acting on BAD [38]. In NPC and GC, ebv-mir-BART11-3p and 5-p induce cancer cell proliferation through the suppression of forkhead box P1 (FOXP1) [39]. Interestingly, ebv-mir-BART9, through the regulation of of E-cadherin expression, has been confirmed to induce epithelial–mesenchymal transition (EMT) and thus promote metastasis [39,40]. In addition, ebv-mir-BART10-3p, through the inhibition of BTRC, essential in the ubiquitination and degradation of β-catenin, induces invasion and metastasis and is thus associated with poor prognosis in NPC patients [41]. Among the mechanisms by which EBV may influence host cell equilibrium, it has been postulated that EBV-infected cells can transfer viral miRNAs via exosomes and thus influence host gene expression in uninfected recipient cells [42]. It has been previously demonstrated that ebv-mir-BART13 can be transferred from B cells to salivary epithelial cells where it downregulates STIM1 protein and decreases activation of NFAT and NFAT-dependent transcriptional activity [43]. In addition, we showed the selective packaging of two v-miRNAs, ebv-mir-BART3 and ebv-mir-BHRF1-1, into the exosomes in a lymphoblastoid cell line [44]. The first, ebv-mir-BART3, addressed importin 7 (IPO7), inducing the pro-inflammatory cytokine IL-6 [24]. The second, ebv-mir-BHRF1-1, downregulated host p53 [45].

**Figure 2:**
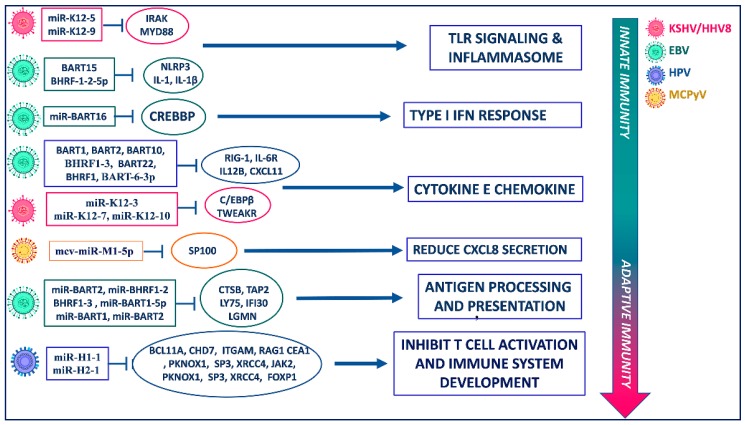
Immunoevasive functions of viral miRNAs. Target cellular components of EBV, KSHV, and HPV miRNAs and the relavant antiviral responses of innate and adaptive immunity.

EBV produces different miRNAs involved in the immunomodulation of both aspecific and specific immunity. Concerning innate immunity, different EBV miRNAs seem to have the ability to influence inflammation and chemotaxis (Figure 2). Ebv-mir-BART6-3p directly binds to RIG-1 mRNA, causing the impaired production of different antiviral cytokines [46]. Moreover, ebv-mir-BART-6-3p, in association with host-derived miR-197, acts on IL-6R mRNA and is involved in the impairment of IL-6 signaling [47]. Host mRNA CREBBP is the target of ebv-mir-BART16 with consequent inhibition of type I interferon signaling [48], which favors the enhancement of viral replication. Ebv-mir-BHRF-1-2-5p acts on interleukin-1 (IL-1) signaling by targeting IL-1 Receptor 1, blocking the activation of host innate immune responses following virus infection [49]. Another EBV-derived miRNA that limits inflammation, an advantage for its own purposes, is ebv-mir-BART15, which regulates both IL-1β production and the NLRP3 inflammasome [50]. Most of the effects of EBV-derived miRNA on the acquired immune system are related to MHC-restricted antigen processing and presentation. Indeed, ebv-mir-BART2 acts on CTSB mRNA and interferes with MHC-I antigen processing, while ebv-mir-BHRF1-3, targeting TAP2, blocks peptide transport to MHC-I [51]. Ebv-mir-BART1-5p also causes an impaired antigen presentation because of its action on LY75 mRNA, which encodes an endocytic receptor involved in antigen capture and processing [52]. Ebv-mir-BART1 and BART2, respectively, act on IFI30 mRNA and LGMN mRNAs, inducing the inhibition of MHC-II-restricted antigen processing [52]. Moreover, EBV-derived miRNA targets genes involved in T cell chemotaxis and polarization; ebv-mir-BART1, -BART2, -BART10, -BART22, and -BHRF1 act on IL12B mRNA preventing the polarization of CD4^+^ T helper cells toward antiviral Th1 subtype [49], while BHRF1-3 targets CXCL11 mRNA, with the consequence of inhibiting the activated T cells’ chemotaxis [53].

Collectively, these studies have highlighted the capability of EBV miRNAs to modulate tumor cell proliferation through complicated regulatory networks including tumor suppressor genes, cell apoptosis, and control of the viral oncogenic protein functions.

## 3. Kaposi’s Sarcoma Herpesvirus/Human Herpesvirus-8 (KSHV/HHV8)

HHV-8, a member of the Herpesviridae family, is an oncogenic virus. HHV-8 shares with EBV the ability to establish a chronic infection in lymphocytes, which are its main reservoir [54], but also in macrophages, keratinocytes, and endothelial cells [55], and to induce cellular transformation [56]. HHV-8 infections are notably threatening in immunocompromised patients, such as those with AIDS or patients with transplants or under chemotherapy treatment [8]. Alongside Kaposi’s sarcoma, from which its alias KSHV (Kaposi’s-sarcoma-associated herpesvirus) was taken [57], HHV-8 is considered the etiological cause of primary effusion lymphoma (PEL) [58] and multicentric Castleman’s disease [59]. HHV-8 has a dsDNA genome encoding for more than 90 open reading frames (ORFs). In addition, HHV-8 encodes for 25 mature miRNAs, deriving from 12 viral pre-miRNAs [60]. All the HHV-8 miRNAs are under the control of latent kaposin promoter. Except for kshv-miR-K10 and kshv-miR-K12, which are expressed more during the lytic phase [61], the majority of the pre-miRNA genes are expressed during the latent phase of virus infection [62] and are located between the sequence for kaposin and open reading frame 71 [62]. As for EBV, HHV-8 latency is the phase of KSHV infection where the v-RNAs cooperate in viral replication and thus contribute to oncogenesis. As an example of this mechanism the KSHV miRNAs kshv-miR-K5, kshv-miR-K7-5p, kshv-miR-K9-5p, kshv-miR-K3, and kshv-miR-K4 have been shown to endorse latency by targeting the KSHV lytic switch protein, either directly or indirectly [63]. The final goals of KSHV miRNAs are immune evasion, avoidance apoptosis, and contribution to tumorigenesis. For example, kshv-miR-K12-1, -3, and -4-3p target and inactivate the inducer of apoptosis, Casp3, blocking apoptosis [64], while kshv-K12-1 functions as an oncogene by activating NF-κB/IL-6/STAT3 signaling [65]. Kshv-mir-K12-3 has been demonstrated to be a promoter of cell migration and invasion by targeting GRK2/CXCR2/AKT signaling [66], and kshv-miR-k12-1-5p has been shown to be a promoter of the proliferation, migration, and invasion of KS cells by suppressing cytokine signaling 6 (SOCS6) [67]. It has also been demonstrated that kshv-miR-K1-5p and kshv-miR-K4-5p directly target CASTOR1, inhibiting its expression and activating the mTORC1 pathway with the final result of promoting tumorigenesis [68]. It has also been shown that kshv-mir-K10a targets tumor necrosis factor-like weak inducer of apoptosis receptor protein (TWEAKR), thus preventing TWEAK-induced apoptosis and inflammatory cytokine (IL8) expression [69]. Interestingly, HHV-8 encodes for miRNAs which share perfect seed homology with cellular oncomiRNAs such as kshv-mir-K12-10, which is a viral orthologue of hsa-mir-142-3p [11]; both miRNAs have been shown to inhibit the TGF-β pathway by targeting the TGF-β type II receptor [70]. Another example of this mechanism comes from kshv-mir-K12-11, a homolog of hsa-mir-155 [71], which targets IKKε, BACH-1, and SMAD5 and downregulates the expression of the basic region/leucine zipper motif transcription factor C/EBPb, a regulator of interleukin-6 [10]. The last known, so far, is kshv-mir-K3, a homolog of hsa-mir-23 with which it shares anti-apoptotic functions by targeting caspase 3 and caspase 7 [12].

Some of the 25 miRNAs encoded by KSHV/HHV8 play an important role in viral latency infection in host cells, targeting key genes and their signaling pathways (Figure 2), interfering with immune surveillance and thus contributing to the development of KS [72]. Two KSHV/HHV8 -derived miRNAs, kshv-mir-K12-5 and kshv-mir-K12-9, affect the secretion of inflammatory cytokines, targeting MYD88 and IRAK1, respectively, which are components of TLR/IL-1R-mediated signaling [73]. Kshv-mir-K12-11 acts on the primary response to antiviral immunity by targeting IKKε with the consequent impairment of type I-IFN signaling [74], while kshv-mir-K12-10 reduces the production of IL-6 and IL-10 by targeting TWEAKR [73]. Kshv-mir-K12-3 and kshv-mir-K12-7, targeting C/EBPβ, modulate cytokine secretion by immune cells such as monocytes or NK lymphocytes [75]. RAB3B and RAB3D are two genes targeted by kshv-mir-K12-3, which not only alter cytokine production, but also attenuate bacterial phagocytosis [76].

## 4. Human Papillomavirus (HPV)

Papillomaviridae is a taxonomic family of non-enveloped DNA tumor viruses that infect both mucosa and cutaneous epithelial cells [77]. To date, 226 genotypes of human papillomavirus (HPV) have been identified [78]. Its circular DNA genome encodes for six nonstructural genes (E1, E2, E4, E5, E6, and E7) and two viral assembly genes (L1 and L2). In particular, E1, E2, and E4 are involved in viral replication, while E5, E6, and E7 are involved in HPV-induced cellular transformation [79,80]. In relation to the ability of HPVs to trigger malignant cellular progression, these viruses are classified as high-risk (HR) and low-risk (LR)-HPVs. LR-HPVs typically cause benign epithelial lesions that may progress to malignant lesions [81], whereas HR-HPVs are associated with cervical carcinomas [82].

Different works have shown that some viruses, including different HPV genotypes, are able to code miRNA-like species, and these miRNAs may be involved in virus-induced carcinogenesis. Unfortunately, very little information is available on the mechanisms by which HPV-encoded miRNAs play a role in the promotion and/or progression of human cancer; this is probably partly due to a lack of proper study models for the various HPV types.

**Figure 3:**
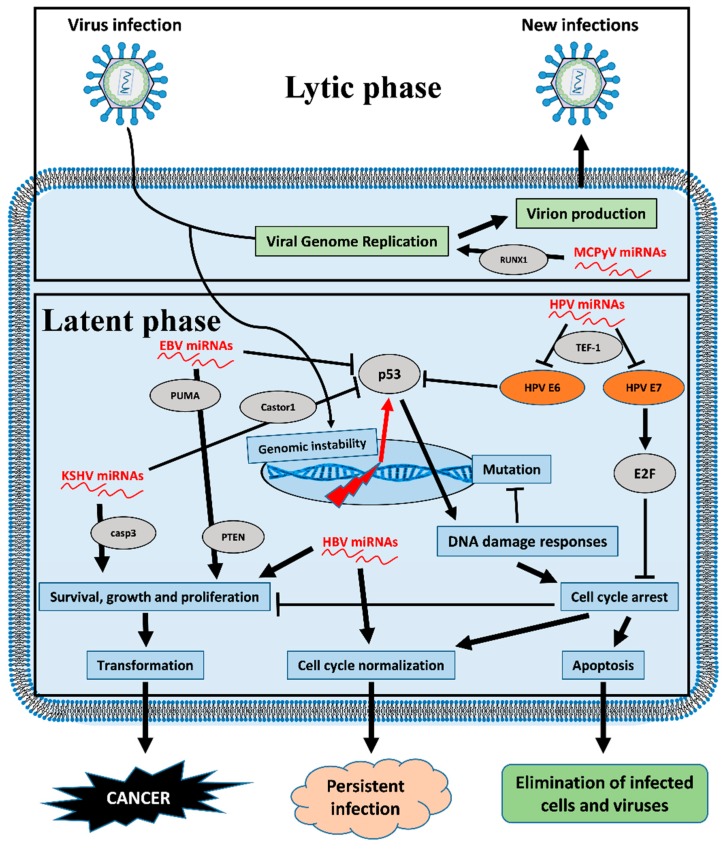
Viral miRNAs affect the pathways involved in the carcinogenesis of host cells. The picture depicts changes to cellular fate because of viral miRNAs. Genomic instability due to viral infection can induce activation of the p53 pathway, which in turn supports both DNA damage responses and cell cycle arrest. In relation to viral infection context, viral miRNAs can affect abortive cell fates such as programmed cell death with consequent cancer transformation. Arrows signify that the factor or process promotes the effect it points to, while blocking arrows signify inhibition. Orange ellipses represent viral proteins, grey ellipses represent host cell proteins, green boxes represent stages of the lytic phase of viral life cycle and blue boxes represent cellular processes affected by viral miRNAs during the viral latent phase.

Recently, to characterize new HPV-encoded miRNAs, Chirayil et al. applied a new approach for miRNA discovery based on forced genome expression. They showed that four different HPV genotypes are mainly involved in the synthesis of miRNAs: HPV17, 37, 41, and a Fringilla coelebs HPV (FcPV1). These data were validated by in vitro assays on cell cultures, and two FcPV1 miRNAs were also found in vivo in a natural host. Interestingly, HPV41-miRNAs and FcPV1-miRNAs are involved in the control of HPV life cycle [83]. In other recent studies, Weng et al. showed that HPV-miRNAs differ in their number depending on the HPV species [15], and Virtanem et al. revealed that miRNAs belonging to HPV-16 species such as miR-H1, miR-H3, miR-H5, and miR-H6 were found in tumor samples [84]. Qian et al. recognized nine putative HPV-encoded miRNAs, (HPV6-mir-H1, HPV16-mir-H1, HPV16-mir-H2, HPV16-mir-H3, HPV16-mir-H5, HPV16-mir-H6, HPV38-mir-H1, HPV45-mir-H1, and HPV68-mir-H1) through tissue sequencing of human cervical lesion and cell lines. These miRNAs are upregulated in chronic infection, interfering with different pathways including regulation of the cell cycle, immune responses, and cell adhesion/migration, and therefore they may be involved in the susceptibility of tissue to transformation [85]. In particular, as depicted in Figure 2, HPV16-derived miRNAs such as miR-H1-1 and miR-H2-1 target different genes involved in immune system regulation. Indeed, miR-H1-1 is able to both inhibit T-cell activation and immune system development, targeting BCL11A, CHD7, ITGAM, RAG1, and TCEA1 genes [85]. Similarly, miR-H2-1 targets different protein involved in both T-cell activation (PKNOX1, SP3, XRCC4) and immune system development (JAK2, PKNOX1, SP3, XRCC4, FOXP1) [85]. Due to the central role of the viral immune escape for the development of virus-related cancers, this could represent a crucial mechanism for HPV-associated cancers. Qian et al. also showed that transcriptional enhancer factor 1 (TEF-1) is a target of HPV-16-encoded miRNAs [85]. Interestingly, this gene regulates cell proliferation and migration, and it binds and activates the early HPV 16 promoter of E6 and E7 [86,87]. E6 is able to induce the degradation of p53 protein [88], while E7 induces the degradation of the retinoblastoma protein, causing release of E2F, with consequent activation of transcription of the target genes and overcoming of proliferation arrest [89]. Therefore, downregulation of E6 and E7 by HPV-encoded miRNA through TEF-1 could thus lead to increased cell cycle arrest of HPV-infected cells for cell cycle normalization controls and persistent HPV infection (Figure 3). These mechanisms could represent indirect processes by which HPV-encoded miRNAs affect tumorigenesis through the controls of major pathways involved in the carcinogenesis of host cells.

Conversely, it has been shown that HR-HPVs are also responsible for both upregulation of oncogenic host miRNAs and downregulation of tumor-suppressive host miRNAs, influencing both viral replication and HPV-induced carcinogenesis [90]. This is because HPVs are able to integrate into the genome at the sites where specific miRNAs are frequently located [90]. Furthermore, the expression of many host miRNAs is regulated by HPV proteins (E6 and E7) and the E2F protein of the host cells [91]. As mentioned above, HPV-16-encoded miRNAs are able to regulate E6, E7, and E2F and, therefore, they can potentially regulate the expression of host miRNAs. Different results obtained using the K14-HPV16 transgenic mice model showed that the expression of deregulated host miRNAs in non-neoplastic samples could regulate the vulnerability to oncogenesis induced by HPV-associated mechanisms [92,93].

Based on current knowledge, the action of HPV-encoded miRNAs in the carcinogenesis process seems to be indirect, regulating, for example, HPV oncogenic proteins such as E6 and E7 which, in turn, regulate the tumorigenesis pathways of host cells. Moreover, HPV-encoded miRNAs could regulate key proteins for inhibition of the immune system allowing viral immune escape and the development of HPV-related cancer.

The characterization of the complete genomes of HPV subtypes by bioinformatics methods also allows the prediction of potential v-miRNAs and their target genes, providing a fundamental tool for understanding the role of HPV-encoded miRNAs in the carcinogenesis process of HPV-associated cancers [15]. Further studies are needed to discover new HPV-encoded miRNAs and their implications for both virus infection and carcinogenesis.

## 5. Hepatitis B Virus (HBV)

In contrast to oncoviruses such as EBV, KSV, and HPV, which dysregulate cellular tumor suppressor activities inducing oncogenicity by affecting p53 and pRB, other viruses do not have a straight correlation with neoplastic transformation. Among them Hepatitis B (HBV) and Hepatitis C viruses (HCV) have been associated, respectively, with 53% and 25% of hepatocellular carcinomas [94]. Hepatocellular carcinoma (HCC) represents the third most common cause of cancer-related death worldwide [95]. Liver cancer is often associated with HBV and/or HCV infection; however, the molecular mechanisms whereby these viruses induce HCC are still not completely elucidated.

HBV belongs to the Hepadnavirus family, and is a hepatotropic virus that possesses a partially double-stranded DNA genome in a relaxed circular DNA (rcDNA) form. This DNA is converted into a covalently closed circular DNA (cccDNA) via the cellular apparatus and organized as a viral minichromosome [96]. Recently, a new viral miRNA involved in HCC was isolated from HBV: HBV-mir-2 [97]. HBV-mir-2 acts in a dual mode by suppressing TRIM35 (tripartite motif containing 35) and stabilizing RAN (ras-related nuclear protein) mRNA by binding to their 3′UTRs [97]. TRIM35 in hematopoietic cells induces apoptosis and inhibits cell proliferation, and for this reason it is described as tumor suppressor [97]. RAN is a protein that is correlated with cell cycle control, nucleocytoplasmic shuttle, and cell transformation. Overexpression in hepatic cell lines (HepG2, Huh7) induces epithelial–mesenchymal transition (EMT), whereas its silencing decreases EMT [97]. RAN is correlated with proliferation, migration, and invasion in ovarian cancer [98] and in HCC [97]. Moreover, these hepatotropic viruses contribute to HCC in an indirect manner by establishing chronic infection; this effect is linked to the long-lasting infection that induces recurrent liver inflammation by the host immune system. In a recent report, it was demonstrated that HBV itself modulates its replicative activity via an endogenous miRNA: HBV-mir-3 [99]. The authors demonstrated that this miRNA represses HBV protein expression and viral production to avoid major damage to the infected cells. In particular, HBV-mir-3 suppresses HBsAg, HBeAg, and HBc protein expression and the intermediate HBV’s DNA replication. The same authors pointed out that the infection persistence has a carcinogenic implication mainly because chronic HBV infection contributes to the occurrence of mutations. In this way, HBV-mir-3 indirectly promotes HCC onset [99].

## 6. Hepatitis C Virus (HCV)

HCV belongs to the Flaviviridae family and it was first discovered in 1989, when a complementary DNA clone encoding for an antigen was associated specifically with NANBH (non-A, non-B hepatitis) infections [100]. It is an RNA virus with a single-stranded positive genome (+) that principally infects hepatocytes [101]. After infection, viral RNA is translated via the cellular ribosomal apparatus and the HCV positive strand is copied, generating a replicative intermediate (RI) with a negative strand (−). One of the mechanisms by which HCV can induce oncogenic transformation is the induction of double strand brakes (DSBs) [102] and oxidative stress [103].

The HCV genome is targeted by cellular miRNA; in particular, has-miR-122, one of the most abundant liver miRNAs, binds two sites (one in the 5′UTR and one in the 3′UTR) in the viral RNA, enhancing its replication [104]. Has-miR-122 inhibition by LNA reduces HCV in vitro and in vivo and has been tested in several clinical trials [105,106], demonstrating the efficacy of this treatment without adverse effects. HCV/has-miR-122 binding does, however, have an effect on the induction of HCC [107]. Luna and coworkers demonstrated that miR-122 co-immunoprecipitates with Ago in association with HCV RNA, which thus acts as a has-miR-122 sponge. It is noteworthy that miR-122 knockout induces liver diseases and HCC [108,109].

Like miR-122, mir-199a* has been described as an HCV genomic RNA binder. Mir-199a* has been demonstrated to inhibit HCV RNA replicative activity by binding a region in the HCV 5′-UTR (domain II of the IRES region), a highly conserved region among the different HCV genotypes [110]. In addition, mir-199a* has been demonstrated to co-immunoprecipitate with Ago2 [110] and to inhibit HCV RNA replicative activity, inducing Ago2 binding to HCV RNA. This mechanism is responsible for a HCV feedback loop that can control the persistence or downmodulation of the virus in the infected cells. In other studies, it has been demonstrated that HCV RNA is not found in cells with HCV-induced mutations, suggesting a “hit and run” mechanism for neoplastic transformation [102]. Surprisingly, and yet to be validated, mir-199a* was recently described as an HCV miRNA by VIRmiRNA, a database for experimentally validated v-miRNAs and their targets [111]. For this reason, miR-199a* could be an interesting objective for targeted HCC therapy.

## 7. Merkel Cell Polyomavirus (MCPyV)

Merkel cell polyomavirus (MCPyV) is a mammalian, double-stranded DNA polyomavirus [112], which causes a lifelong but inoffensive infection [113]. In immunosuppressed patients, such as solid-organ-transplanted or autoimmune-condition-affected patients, the presence of MCPyV increases the occurrence of Merkel cell carcinoma, a neuroendocrine skin cancer [114,115]. The MCPyV genome is long (5386 bp) and encodes for two early antigens, long LT and small sT-Ag, and two late structural antigens, VP1 and VP2 [116,117]. In addition, it has been reported that two miRNAs, mcv-miR-M1-5p and -3p, edited from a single miRNA precursor [118], are the only miRNAs expressed by actively replicating MCPyV genomes [119]. The miRNA precursor is expressed from the antisense strand of the LT ORF and shows perfect sequence complementarity to a region in exon 2 of the MCPyV LT mRNA transcript [120]. The first evidence for the roles played by mcv-miR-M1 miRNAs indicates the capability to autoregulate early viral gene expression at late stages post infection [118]. The demonstrated regulation of expression of the large T-antigen could potentially lead to the evasion of immune surveillance [118]. Akhbari et al. ran an in silico analysis of the mcv-miR-M1-5p seed sequence and found the direct targeting of SP100, an intrinsic antiviral protein, leading to a reduction in the secretion of CXCL8 with a final effect of the subversion of the host-cell immune response [120]. By analyzing the seed region, Lee at al. built a list of predicted human target genes of the experimentally observed mature mcv-miR-M1 which could be relevant for tumorigenesis processes [121]. Predicted targets include PIK3CD and PSME3, responsible for antigen presentation by the host cell [122,123], which are potentially involved in mediating the host immune response against MCPyV [121]. Another predicted target of mcv-miR-M1 is RUNX1, a transcription factor known to play the roles of an oncogene and an anti-oncogene in epithelial tumors. By downregulating RUNX1, it has been suggested that mcv-miR-M1 could aid the viral life cycle transition from early to late [121] and affect polyomavirus replication [124].

## 8. Conclusions

Viruses have developed a complex symbiotic system by which to access and regulate host transcriptional machinery. Among these, the discovery of the first miRNAs coded by a virus by Pfeffer et al. [23] paved the way for a very new field of interest: viral mechanism interpretation. Viral miRNAs are ideal tools, because of their non-immunogenicity, to induce immune invisibility of infected cells [8]. The best-known and studied viral miRNAs are the herpetic ones, as is their transcriptional process. Less is known about newly discovered miRNAs, such as for HBV, HVC, and MCPyV, but this fascinating and new mechanism of transcription regulation will surely be the subject of further studies.

In general, viral latency is the phase in which the majority of v-miRNAs are transcribed, serving as an immune evasion strategy and thus regulating host processes in order to promote cell survival. The occurrence of cancers driven by so-called oncoviruses must be considered an unexpected, unfortunate side effect of the infection itself, decreasing transmissibility and replication fitness. In the case of immunosuppressed populations, cancers caused by tumor viruses have an even more increased occurrence [3] suggesting the deep relationship between viruses and the immune system. In this review, we focused our attention on the miRNAs encoded by the viruses EBV, KSHV/HHV-8, HPV, HBV, HCV, and MCPyV These viruses are been related to cancer occurrence in many different populations, but especially in immunosuppressed populations such as HIV or transplant patients. Recently, it has been shown that these viruses produce different oncoviral proteins that cause functional impairment of p53 activity, which is a crucial mechanism of virus-related carcinogenesis [125]. Interestingly, v-miRNAs also seem to have p53 as the main target, which therefore becomes an even more central mechanism by which these viruses can induce oncogenetic processes (Figure 3).

Due to v-miRNA effects, ranging from increasing viral proliferation and increased virulence to tuning the host immune responses, it is not surprising that viral miRNA expression shows great therapeutic potential and represents an appealing antiviral strategy for the miRNA-based treatment of viral infections. Moreover, v-mirRNAs are now the most promising tool for measuring virus infective and reproductive status, with significant value as diagnostic and prognostic biomarkers. Once the mechanism behind v-miRNA actions is more elucidated and clarified, they may really be used for the early diagnosis of virus-related tumors.

## Figures and Tables

**Table 1:** Overview of viral miRNA regulatory functions and targets.

VIRUS	miRNA	Targets	Effects of miRNAs
EBV	ebv-BHRF1-2	IL-12, CATHEPSIN B, AEP, GILT	Immune evasion
	ebv-BHRF1-3	BHRF1-3, TAP	
	ebv-BART1-5p	IL12, CATHEPSIN B, AEP, GILT	
	ebv-BART2-5p	MICB, IL-12, CATHEPSIN B, AEP, GILT	
	ebv-BART3-3p	IPO7	
	ebv-BART5-5p	LMP1	
	ebv-BART6-3p	RIG-1	
	ebv-BART15	NLRP3	
	ebv-BART16	CREB-BP	
	ebv-BART17-5p	TAP	
	ebv-BART22	LMP2A	
	ebv-BHRF1-1	P53	Anti-apoptosis
	ebv-BHRF1-2	PRDM1/Blimp1	
	ebv-BHRF1-3	PTEN	
	ebv-BART1-3p	CASP3	
	ebv-BART4-5p	Bid	
	ebv-BART5-5p	PUMA	
	ebv-BART6-5p	OCT1	
	ebv-BART8	STAT1	
	ebv-BART13-3p	CAPRIN2	
	ebv-BART16	CREB-BP, TOMM22, CASP3, SH2B3	
	ebv-BART22	MAP3K5, CASP3, PAK2, TP53INP1	
	ebv-BART22	NDRG1	Promote metastasis
	ebv-BHRF1-1	RNF4	Promote viral production
	ebv-BHRF1-2	BHRF1	Maintain latency
	ebv-BART2-5p	BALF5	
	ebv-BART6-5p	DICER	
	ebv-BART18-5p	MAP3K2	
	ebv-BART20-5p	BZLF1, BRLF1	
	ebv-BART1-5p	LMP1	Promote cancer development
	ebv-BART16	LMP1	
	ebv-BART17-5p	LMP1	
	ebv-BART1-5p	PTEN	Promote tumor metastasis
	ebv-BART7-3p	PTEN	
	ebv-BART9	E-Cadherin	
	ebv-BART10-3p	BTRC	
	ebv-BART6-3p	PTEN	Promote proliferation
	ebv-BART11	FOXP1	
KSHV	kshv-miR-K12-1	Casp3	Apoptosis
	kshv-miR-K12-3	Casp3	
	kshv-miR-K12-4	Casp4	
	kshv-miR-K12-5	Tmskα1	
	kshv-miR-K12-10a	TWEAK	
	kshv-miR-K12-12	CASP3, CASP7	
	kshv-miR-K12-1	NF-κB signaling/IκBα	KSHV latency
	kshv-miR-K12-3	nuclear factor I/B, GRK2	
	kshv-miR-K12-4	Rbl2	
	kshv-miR-K12-7	RTA (KSHV ORF50)	
	kshv-miR-K12-9	RTA (KSHV ORF50), BCLAF1	
	kshv-miR-K12-10a	BCLAF1	
	kshv-miR-K12-11	MYB, IKKε	
	kshv-miR-K12-1	THBS1	Cell adhesion, migration, and angiogenesis
	kshv-miR-K12-3	THBS1	
	kshv-miR-K12-6	THBS1, Bcr, SH3BGR	
	kshv-miR-K12-11	THBS1	
	kshv-miR-K12-1	CASTOR1, STAT3,p21	Promote tumorigenesis, Cell survival
	kshv-miR-K12-4	CASTOR1	
	kshv-miR-K12-10a	TGFBR2	
	kshv-miR-K12-10b	TGFBR2	
	kshv-miR-K12-11	SMAD5	
	kshv-miR-K12-1	MICB	Immune evasion
	kshv-miR-K12-3	C/EBPβ p20 (LIP)	
	kshv-miR-K12-5	MYD88	
	kshv-miR-K12-7	C/EBPβ p20 (LIP), MICB	
	kshv-miR-K12-9	IRAK1	
	kshv-miR-K12-11	C/EBPβ	
	kshv-miR-K12-1	MAF	Differentiation of infected cells
	kshv-miR-K12-6	MAF	
	kshv-miR-K12-11	MAF/BACH-1	
HPV	HPV16-miR-H1	BCL11A, CHD7, ITGAM, RAG1, TCEA1	Immune evasion
	HPV16-miR-H2	SP3, XRCC4, JAK2, PKNOX1, FOXP1	
HBV	HBV-mir-2	TRIM35	Promote tumorigenesis
	HBV-mir-3	HBsAg, HBeAg, HBc	Self-replication
MCPyV	MCV-miR-M1-5p	SP100	Immune evasion
	MCV-miR-M1	RUNX1	Viral proliferation
